# Opening of the Medical Schools

**Published:** 1880-11

**Authors:** 


OPENING OF THE MEDICAL SCHOOLS.—We have learned, thus far only, that the Medical Schools of Philadelphia have their usual full classes, and that the prospects of the University of Md. Medical School of this City, are very encouraging for a large class. These are all good institutions, and worthy the patronage they severally receive. If young men *will* study medicine, it is far better they should seek the *best* and most reputable schools, even if they have to pay a little more fees! And it would be well for them to bear in mind that the dearest articles, and a medical diploma is no exception, are the cheapest in the long run. Cheap medical schools are like cheap doctors, and many other cheap things that *are not* more valuable than the estimate *they place upon themselves*, in under bidding in the race for public patronage and favor.

The reasonable inference, however, is that the better class of students will always seek the older, more reputable and thorough institutions, of which there are a number; and the others the "Cheap John" establishments, which, unfortunately for the profession, are sufficiently numerous to meet all the demands that are made upon them. These will doubtless continue work, "*non missura cutem, nisi plena cruoris, hirudo*," or as long as Cheap Johnism pays.

